# Rapid Pain Reduction Following a Multimodal Physiotherapy Protocol in a Retrospective Case Series of Patients With Persistent Scrotal Content Pain

**DOI:** 10.7759/cureus.113378

**Published:** 2026-07-25

**Authors:** Christopher Mendias, Tariq M Awan

**Affiliations:** 1 Research Division, Performance Medicine Institute, Phoenix, USA

**Keywords:** orchialgia, pain management, pelvic floor therapy, physical therapy, physiotherapy, scrotal content pain, scrotal pain, tadalafil

## Abstract

Introduction: Scrotal content pain (SCP) is a clinically significant condition that can substantially impair quality of life. The causes of SCP are heterogeneous and may include urologic, neuropathic, musculoskeletal, vascular, inflammatory, referred, and psychosocial contributors. In some patients, dysfunction of the lower abdominal wall, inguinal canal, or pelvic floor may contribute to regional nerve irritation and persistent pain. The objective of this retrospective case series was to describe pain and quality-of-life outcomes following a multimodal physiotherapy protocol in men with persistent SCP.

Methods: Twenty-eight men with persistent SCP, with a mean age of 42.1 years (95% CI, 36.3 to 47.9), who completed the four-week protocol and had complete baseline and post-treatment outcome data were included. The protocol consisted of four weekly sessions of class IV laser therapy, dry needling, pulsed electromagnetic field therapy, manual therapy, progressive therapeutic exercise, and daily oral tadalafil. Pain was measured before and after each session using a 10-cm visual analog scale (VAS). Physical and mental health were assessed before treatment and after the final session using the Patient-Reported Outcomes Measurement Information System (PROMIS) Global-10. Available follow-up data were reviewed for symptom recurrence and additional treatment.

Results: Mean VAS pain decreased from 8.0 cm (95% CI, 7.8 to 8.2) before the first treatment session to 3.0 cm (95% CI, 2.6 to 3.4) immediately after the first session and to 0.8 cm (95% CI, 0.4 to 1.1) after the fourth session. PROMIS Global-10 Physical Health scores increased from 40.6 to 51.7, and Mental Health scores increased from 46.7 to 51.1. At three to six months, 25 patients were contacted, and none reported pain recurrence requiring additional treatment. At six to 12 months, 22 patients were contacted, of whom four reported recurrence that interfered with activities of daily living (ADL) and underwent additional treatment.

Conclusions: In this retrospective case series, completion of a four-week multimodal protocol was associated with substantial short-term reductions in self-reported pain and improvements in global physical and mental health in men with persistent SCP. Because the study lacked a control group, included multiple concurrent interventions, and had incomplete long-term follow-up, causality, component-specific effects, and treatment durability cannot be established. Prospective controlled studies are warranted.

## Introduction

Scrotal content pain (SCP) represents a significant clinical challenge. Approximately 1% of men experience chronic SCP, and symptoms may substantially interfere with physical activity, sexual function, work, sleep, and other activities of daily living (ADL) [1,2]. The causes of SCP are heterogeneous and may include infection, inflammation, tumor, varicocele, hydrocele, prior surgery, vascular abnormalities, neuropathic pain, musculoskeletal dysfunction, referred pain, and psychosocial factors [1-3]. In some patients, no clear structural or infectious cause can be identified despite clinical evaluation. Conservative treatments, including anti-inflammatory medications, neuropathic pain medications, pelvic floor physiotherapy, and spermatic cord blocks, may provide relief, although treatment responses are variable and may be temporary [1,2]. When conservative treatment fails, microsurgical denervation of the spermatic cord or, less commonly, orchiectomy may be considered. These procedures are invasive and may be associated with complications, including testicular atrophy, hydrocele, sensory changes, and persistent pain [1,2].

Musculoskeletal and neuropathic contributors may be clinically relevant in a subset of patients with SCP. Musculoskeletal etiologies have been identified in up to 39% of patients presenting with SCP, and some patients do not have reproducible tenderness of the testicle or epididymis on examination [3]. Referred pain may arise from the lumbar spine, hip, abdominal wall, inguinal canal, pelvic floor, or regional peripheral nerves. The scrotum and spermatic cord receive sensory innervation from the genitofemoral, ilioinguinal, and pudendal nerves. Histologic evaluation of spermatic cords from men undergoing denervation for chronic pain has demonstrated degenerative changes in small nerve fibers within the cremasteric muscle and spermatic cord fascia [4]. These findings support a possible neuropathic component in some patients, although the relationship between nerve pathology, myofascial dysfunction, vascular factors, and clinical symptoms remains incompletely understood. Increased tone or restriction involving the cremaster muscle, abdominal wall, inguinal canal, or pelvic floor may contribute to regional nerve irritation and perpetuation of pain in some patients. Persistent nociceptive input may also promote protective muscle activation, pain-related fear, and altered movement patterns, creating a possible pain-spasm cycle [5].

The heterogeneous and often multifactorial nature of persistent SCP creates a need for conservative treatment strategies that can address several potential contributors simultaneously. Physiotherapy interventions may be particularly relevant when pain is associated with myofascial restriction, altered neuromuscular control, regional nerve irritation, or impaired movement patterns. We therefore selected established physiotherapy approaches that have demonstrated analgesic, neuromuscular, or tissue-modulating effects in other musculoskeletal and pelvic pain conditions and applied them as a bundled protocol for persistent SCP. High-intensity class IV laser therapy has been used as an adjunctive treatment for pain and functional impairment in patients with musculoskeletal disorders, although treatment protocols and the certainty of the evidence vary across conditions [6]. Preclinical models suggest that class IV laser therapy may inhibit nociceptive signaling involving transient receptor potential vanilloid (TRPV) channels in Aδ and C nociceptive fibers without measurably impairing motor function [7,8]. Laser therapy may therefore offer a theoretical advantage over local anesthetics, which broadly inhibit both sensory and motor nerve function, by creating a temporary, motor-sparing analgesic window for manual therapy and active rehabilitation. Dry needling and manual therapy were selected based on evidence that these interventions may reduce myofascial pain and tissue restriction [9,10]. Pulsed electromagnetic field therapy was included for its potential analgesic and tissue-modulating effects, while progressive therapeutic exercise and neuromuscular stimulation were selected based on their potential to improve lumbopelvic and abdominal neuromuscular control [11-15]. Additionally, tadalafil has demonstrated clinical benefits in men with chronic pelvic pain syndrome and was incorporated as a pharmacologic adjunct to the protocol [16]. The objective of this retrospective case series was to describe changes in pain and health-related quality of life following the four-week multimodal protocol and to summarize available follow-up data regarding symptom recurrence.

## Materials and methods

Participants

This retrospective case series included adult men treated for persistent SCP at the Performance Medicine Institute (Phoenix, AZ), with initial evaluation and treatment between January 2024 and March 2025. Patients were eligible for inclusion if they had experienced persistent SCP for at least three weeks, completed the four-week multimodal treatment protocol, and had complete baseline and post-treatment patient-reported outcome measures. All patients meeting these criteria during the study period were included in the analysis. This study was conducted in accordance with the Declaration of Helsinki. Pearl IRB (Fishers, IN) determined that this study was exempt under 45 CFR 46.104(d)(4), Secondary Research Uses of Data or Specimens (IRB Protocol #2026-0350).

Cases were identified through retrospective review of the electronic medical record. Before treatment, patients underwent a clinical evaluation by the treating clinician that included vital signs, a medical and genitourinary history, examination of the scrotal contents and inguinal region, and assessment for potential musculoskeletal or referred contributors to pain. The examination routinely included palpation of the testicle, epididymis, spermatic cord, inguinal canal, abdominal wall, and external pelvic floor to identify tenderness or reproduce symptoms. The lumbar spine and hip were also evaluated from a musculoskeletal perspective. Scrotal ultrasonography was obtained when hydrocele, varicocele, or another structural abnormality was suspected. No included patient had clinical findings that raised concern for an active genitourinary infection. When infection or systemic inflammation was considered in the differential diagnosis, laboratory evaluation included high-sensitivity C-reactive protein and erythrocyte sedimentation rate, with additional testing or urologic referral performed as clinically indicated. When prostatitis or obstructive urinary symptoms were suspected, prostate-specific antigen testing and additional clinical evaluation were performed as indicated. Alternative diagnoses were determined by the treating clinician based on the history, physical examination, laboratory findings, imaging, and specialist evaluation when obtained.

Records were excluded when symptoms were better explained by another diagnosis, including inguinal hernia, tumor, clinically significant varicocele requiring primary urologic management, prostatitis, urinary tract infection, sexually transmitted infection, or another active genitourinary infection. Patients with acute scrotal pain requiring urgent urologic evaluation were excluded. Patients with hypotension or another contraindication to tadalafil were not eligible for the bundled protocol. Prior antibiotic treatment without objective evidence of infection was not considered a reason for exclusion. Treatment-related adverse events documented during the four-week protocol were extracted from the clinical record.

Treatment

**Figure 1:**
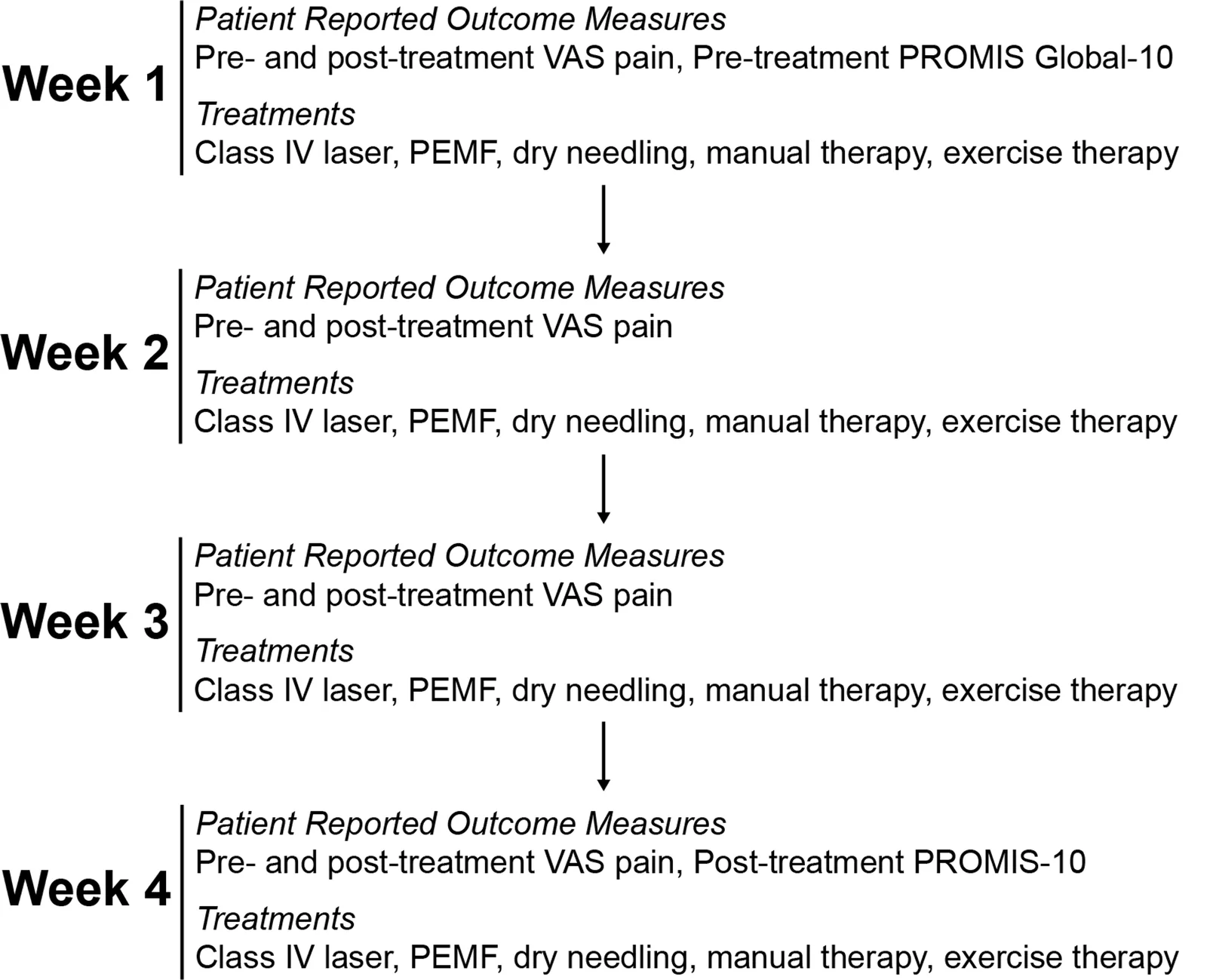
Overview of study timeline and measures PEMF: pulsed electromagnetic field; VAS: visual analog scale; PROMIS: Patient-Reported Outcomes Measurement Information System

The intervention was delivered as a bundled clinical protocol, and the study was not designed to determine the independent contribution of any individual treatment component. Patients underwent four weekly treatment sessions consisting of class IV laser therapy, dry needling, pulsed electromagnetic field therapy, manual therapy, progressive therapeutic exercise, and daily oral tadalafil (Figure 1).

**Figure 2:**
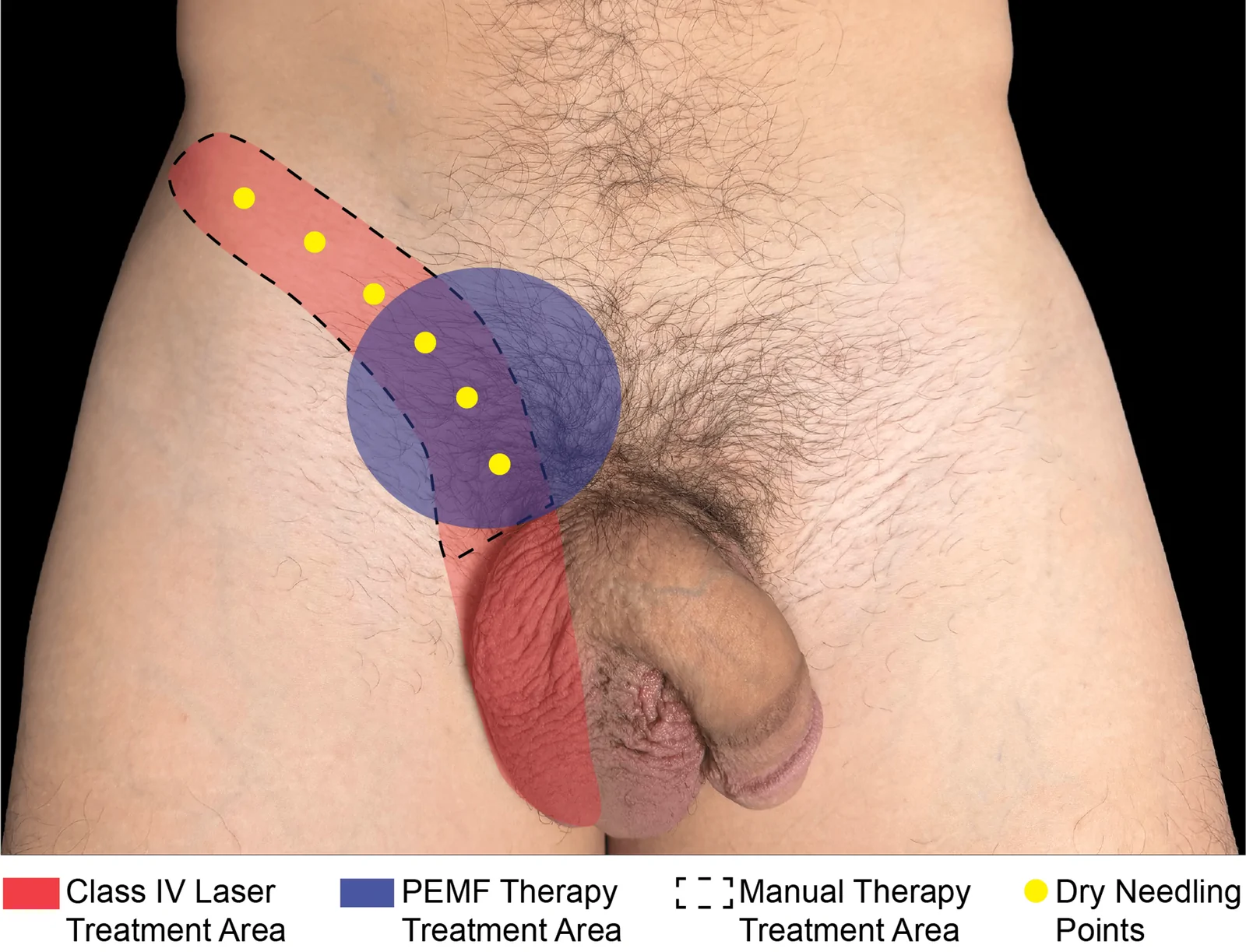
Representative treatment intervention sites PEMF: pulsed electromagnetic field Informed consent was obtained from the patient for publication of this image.

Photobiomodulation was delivered using a class IV laser system (REMY Medical Laser, Alpharetta, GA) in dual-wavelength mode at 810 nm and 980 nm. Treatment was applied using a handheld applicator in continuous scanning mode from the proximal inguinal canal to the distal scrotal region (Figure 2). The treatment protocol delivered a total energy dose of 800 to 1000 J per session. Dry needling was performed using 0.18 mm × 15 mm needles (SERIN, Boston, MA). Approximately six needles were placed intradermally or subcutaneously over the inguinal canal and adjacent treatment region (Figure 2) and retained in place for 15 to 20 minutes. Pulsed electromagnetic field therapy (BioElectronics, Frederick, MD) was delivered using a fixed protocol for 20 to 30 minutes over the external abdominal ring and cremasteric region (Figure 2).

Manual therapy included myofascial release, soft tissue mobilization, proprioceptive neuromuscular facilitation stretching of the lumbopelvic complex, and low-grade mobilization of the hip or sacroiliac joint when indicated by examination findings and symptoms. Therapeutic exercises were selected according to baseline activity level, fitness, mobility, symptom tolerance, and neuromuscular deficits. Exercise progression followed standard neuromuscular and progressive overload principles, while the specific exercises, volume, and rate of progression were individualized because of variations in baseline functional capacity. Exercise progression generally began with foundational exercises (isometric adduction, supine march, pelvic tilts, cat-cow, quadruped rocking, and glute bridge), progressed to intermediate exercises (single-leg bridge, double-leg raise, step-downs, Swiss ball rollouts, side-lying abduction, dead bugs, squats, Pallof press, and body blade rotations), and then advanced to advanced exercises (Copenhagen plank, reverse lunges, reverse deadlifts, Bulgarian split squats, Russian twists, and bicycle crunches). Direct-current neuromuscular stimulation (Neubie, NeuFit, Austin, TX) was used selectively in patients with more pronounced neuromuscular inhibition, with electrode placement and stimulation intensity adjusted according to treatment goals and patient tolerance. Patients were instructed to perform exercises and stretching at home two to three times weekly, along with diaphragmatic breathing. All patients were prescribed tadalafil 5 mg daily throughout the four-week protocol, and adherence was reviewed at each treatment visit.

Patient-reported outcome measures and follow-up

Pain intensity was assessed using a 10 cm visual analog scale (VAS), with 0 cm representing "no pain" and 10 cm representing the "worst pain imaginable." Patients completed the VAS immediately before and after each of the four weekly treatment sessions. The pre-treatment VAS measurement from the first session was considered the baseline pain score, and the post-treatment VAS measurement from the fourth session was considered the final treatment-period pain score.

Health-related quality of life was assessed using the National Institutes of Health (NIH) PROMIS Global-10 questionnaire [17]. Patients completed the PROMIS Global-10 before the first treatment session and after the fourth treatment session. Responses were scored according to the published PROMIS scoring method and converted to Global Physical Health and Global Mental Health T-scores, with a population mean of 50 and a standard deviation of 10. Higher scores indicated better self-reported health.

Follow-up

Available follow-up information was collected by telephone, short message service (SMS) messaging, or during subsequent clinic visits at approximately three to six months and six to 12 months after completion of the protocol. Patients were asked about satisfaction with treatment, recurrence of SCP, interference with ADL, and the need for additional treatment. A clinically relevant recurrence was defined as recurrent pain that interfered with ADL and prompted the patient to undergo additional treatment. For patients who underwent retreatment, VAS pain was recorded before and after the additional treatment course, along with the number of treatment sessions required.

Data availability

Data are available upon reasonable request from the corresponding author.

Statistics

VAS pain scores were analyzed using a two-way repeated-measures analysis of variance, with treatment week and within-session time point as repeated factors. The interaction between treatment week and within-session time point was also evaluated. Tukey-adjusted multiple-comparison tests were used to compare each Week 2 through Week 4 pre-treatment score with the Week 1 pre-treatment baseline, each post-treatment score with the Week 1 pre-treatment baseline, and each post-treatment score with the corresponding pre-treatment score from the same week. PROMIS Global Physical Health and Global Mental Health T-scores were compared between baseline and Week 4 using paired t-tests. Statistical tests were two-sided, and an alpha level of 0.05 was considered statistically significant.

The distributions of model residuals were examined for approximate normality. Sphericity was assessed for repeated factors with more than two levels, and the Greenhouse-Geisser correction was applied when the sphericity assumption was not met. Values are presented as means with 95% confidence intervals, reported as (lower 95% confidence limit, upper 95% confidence limit), unless otherwise indicated.

## Results

**Figure 3:**
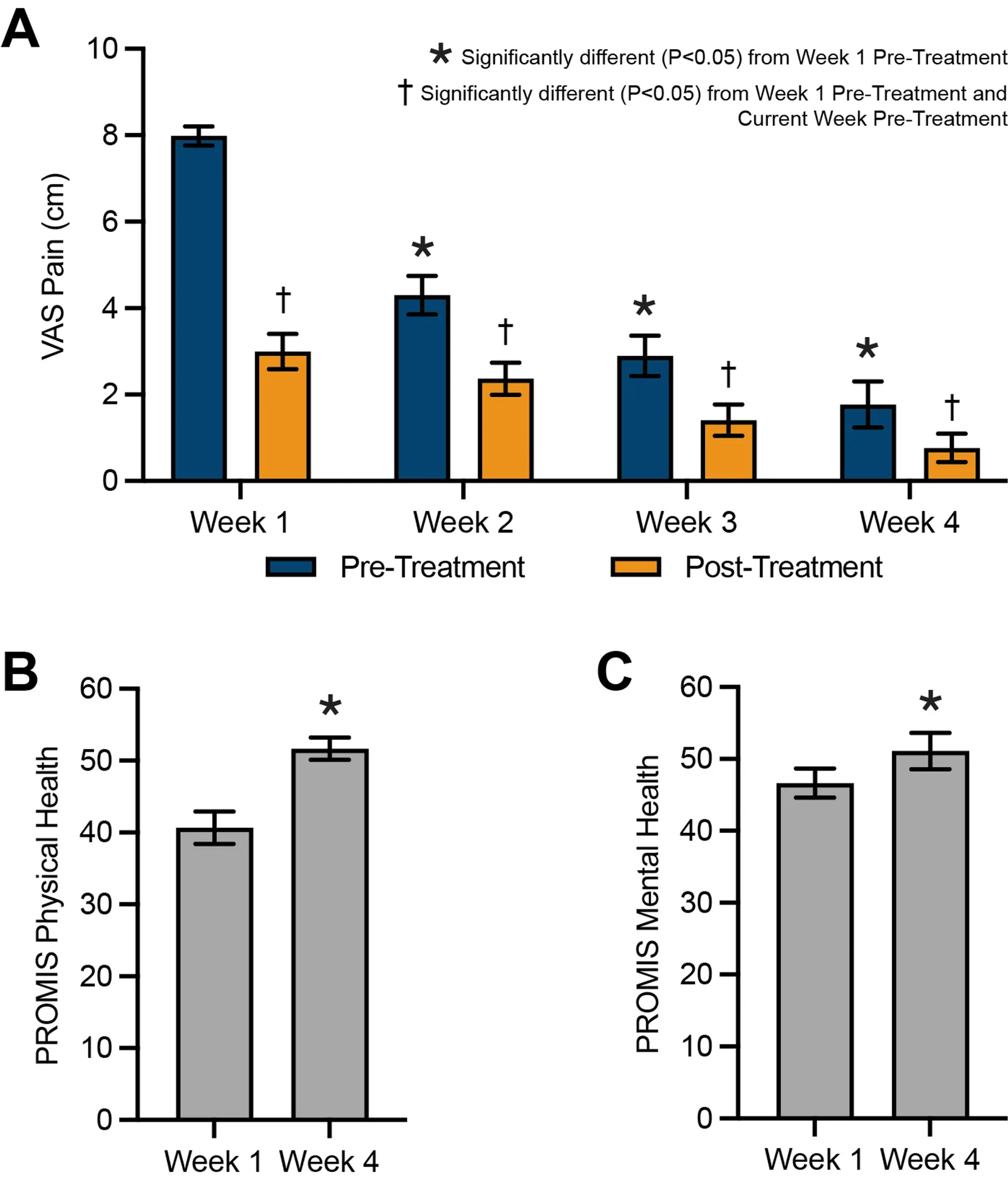
Patient-reported outcome measures Patient-reported outcome measures. (A) Changes in VAS Pain scores over time. Differences were analyzed using a two-way repeated-measures ANOVA with Tukey-adjusted multiple-comparison tests. (B) PROMIS Global-10 Physical Health and (C) PROMIS Global-10 Mental Health scores. Differences were analyzed using paired t-tests. Values are presented as means with 95% confidence intervals. N=28 patients. VAS: visual analog scale; PROMIS: Patient-Reported Outcomes Measurement Information System; ANOVA: analysis of variance

Twenty-eight men met the inclusion criteria and were included in the analysis. Mean age was 42.1 years (36.3, 47.9), and mean symptom duration before treatment was 3.5 months (3.0, 4.0). Pain scores decreased, and quality-of-life scores increased over the four-week treatment period (Figure 3). Mean VAS pain decreased by 63% from 8.0 cm (7.8, 8.2) before the first treatment session to 3.0 cm (2.6, 3.4) immediately after the first session (P<0.05). Pain scores continued to decrease across the four-week protocol, reaching 0.8 cm (0.4, 1.1) after the final treatment session (P<0.05), representing a 90% reduction from baseline (Figure 3A). PROMIS Global-10 Physical Health scores increased by 27% from 40.6 (38.3, 42.9) at baseline to 51.7 (50.1, 53.2) at Week 4 (P<0.05, Figure 3B). PROMIS Global-10 Mental Health scores increased by 9% from 46.7 (44.6, 48.7)to 51.1 (48.6, 53.6) over the same period (P<0.05, Figure 3C).

Follow-up outcomes are presented in Table 1. At three to six months after completion of treatment, 25 of 28 patients were successfully contacted. All 25 respondents reported satisfaction with the treatment outcome, and none reported recurrent pain that interfered with ADL or required additional treatment. At six to 12 months, 22 of 28 patients were successfully contacted. Four of the 22 respondents reported recurrent pain that interfered with ADL and underwent additional treatment. All four patients reported that symptom recurrence occurred during periods of increased emotional stress, although no causal relationship between stress and recurrence could be established. Mean VAS pain at recurrence was 6.1 cm (4.1, 8.1) and decreased by 75% to 1.5 cm (0.8, 2.3) after retreatment. The mean number of treatment sessions required for recurrent symptoms was three (2, 4).

**Table 1:** Treatment follow-up Number of subjects who were reachable, who had a sustained response, and who had an ADL-interfering recurrence requiring treatment. The four patients with recurrence underwent a repeat course of the same protocol. VAS pain score values are presented as mean (lower 95% confidence interval, upper 95% confidence interval). ADL: activities of daily living; VAS: visual analog scale

Follow-up window	Reachable (N)	Sustained response (N)	ADL-interfering recurrence requiring treatment (N)	VAS pain at recurrence (cm)	VAS pain after treatment of recurrence (cm)	Treatment sessions for recurrence
3 to 6 months	25	25	0	-	-	-
6 to 12 months	22	18	4	6.1 (4.1, 8.1)	1.5 (0.8, 2.3)	3 (2, 4)

Four patients reported mild headaches associated with tadalafil use. The headaches resolved within one to two weeks while the patients continued tadalafil, and no patient discontinued the medication because of headache. No serious treatment-related adverse events were documented.

## Discussion

This retrospective case series found substantial reductions in self-reported pain and improvements in health-related quality of life following a four-week multimodal protocol for persistent SCP. Mean VAS pain decreased by 63% after the first treatment session and by 90% from baseline after the final session. The magnitude of pain reduction exceeded the 30% to 50% improvement threshold proposed for urological chronic pelvic pain cohorts [18]. Improvements were also observed in PROMIS Global Physical Health and Mental Health scores. Among the 22 patients contacted at six to 12 months, four reported recurrent pain that interfered with ADL and underwent additional treatment. These findings are clinically notable in the context of SCP, for which conservative treatments such as anti-inflammatory medications and spermatic cord blocks may provide inconsistent or temporary relief [1,2]. Microsurgical denervation can reduce pain in selected patients but is irreversible and carries risks that include testicular atrophy, hydrocele, and permanent sensory changes [19]. However, direct comparison between the present findings and published surgical outcomes is not possible because of differences in patient selection, symptom severity, treatment history, outcome definitions, and follow-up duration. The present results should therefore be interpreted as preliminary evidence supporting further evaluation of a multimodal conservative approach rather than evidence of equivalence or superiority to surgical treatment. 

One possible explanation for the rapid reduction in pain is the motor-sparing analgesic rationale underlying the protocol. Preclinical studies suggest that photobiomodulation can inhibit nociceptive signaling involving TRPV1-expressing C and Aδ fibers, reduce nociceptive mediator expression, and suppress activity in superficial dorsal horn regions without measurably impairing motor output or deeper tactile and proprioceptive signaling [7,8,20-22]. These findings support the hypothesis that class IV laser therapy may temporarily reduce nociceptive input while preserving the motor function needed for manual therapy and active rehabilitation. Persistent nociceptive input can contribute to peripheral and central sensitization, altered motor responses, and protective muscle activation [5,15]. A temporary reduction in nociceptive signaling, combined with immediate participation in therapeutic exercise and manual therapy, could therefore facilitate desensitization and restoration of more normal lumbopelvic and abdominal motor control. However, the present study did not include neurophysiological, sensory, imaging, or electromyographic measures, so TRPV1 modulation, fiber-selective analgesia, reversal of central sensitization, and changes in cremasteric or pelvic floor motor control remain plausible mechanisms rather than demonstrated effects of the protocol.

The remaining components of the protocol were selected to address potentially overlapping myofascial, inflammatory, neuromuscular, and neurovascular contributors to pain. Dry needling may reduce myofascial pain and alter nociceptive processing, while manual therapy may help address muscular trigger points and connective tissue restrictions in the abdominal, pelvic, and hip regions [9,10]. Pulsed electromagnetic field therapy has demonstrated analgesic, anti-inflammatory, and tissue-modulating effects in other musculoskeletal conditions [11,12]. Progressive therapeutic exercise and neuromuscular stimulation were used to improve movement tolerance, muscle activation, and lumbopelvic control, with exercise selection individualized according to baseline function and symptom response [13-15]. Tadalafil was incorporated as an additional component of the comprehensive protocol based on experimental evidence of antinociceptive, vascular, and peripheral nerve effects [23,24] and clinical evidence of improvements in overall symptoms and quality of life among men with chronic pelvic pain syndrome [16]. Together, these interventions provided a biologically plausible approach for addressing several potentially interacting contributors to SCP. However, because the components were administered concurrently, the present study cannot determine the independent contribution or necessity of any individual modality.

The recurrence findings also suggest that longer-term outcomes may be influenced by factors beyond local tissue and neural mechanisms. Four patients reported recurrence during periods of increased emotional stress, although the observational nature of these reports does not establish a causal relationship. Chronic pain reflects interactions among nociceptive processing, psychological factors, protective behavior, and changes in movement and activity [5,14,15]. The improvements observed in pain and PROMIS scores may therefore reflect changes across several domains of health and function rather than a single peripheral mechanism. Future prospective studies should include measures of psychological distress, pain-related fear, sleep, physical activity, and participation in daily activities in addition to pain intensity and physical examination findings.

This study has several limitations. The retrospective, single-center design and modest sample size limit generalizability and do not permit causal inference. Because the study lacked a sham-treated or comparator group, spontaneous improvement, regression to the mean, placebo and contextual effects, expectation bias, and natural fluctuations in pain cannot be excluded. Treatment was not blinded, and outcome collection may have been influenced by patient expectations or clinician involvement in care. Inclusion was limited to patients who completed the four-week protocol and had complete patient-reported outcome data, which may have introduced selection bias if patients who discontinued treatment or had incomplete assessments experienced different outcomes. The intervention was intentionally delivered as a comprehensive bundled protocol, but the concurrent use of class IV laser therapy, dry needling, pulsed electromagnetic field therapy, manual therapy, therapeutic exercise, neuromuscular stimulation, and tadalafil prevents determination of the independent contribution or necessity of any individual component. Tadalafil was included as a pharmacological component of the protocol based on experimental antinociceptive and neurovascular evidence and clinical evidence in male chronic pelvic pain syndromes. However, the present study cannot determine how much of the observed improvement was associated with tadalafil, the physiotherapy components, their combined use, or other contextual factors. The proposed mechanisms involving fiber-selective photobiomodulation, central sensitization, myofascial dysfunction, neuromuscular re-education, and neurovascular support were not directly measured and therefore remain inferential. Follow-up was incomplete, with 25 of 28 patients contacted at three to six months and 22 of 28 contacted at six to 12 months. Nonresponders may have experienced different outcomes, and the available follow-up data may therefore overestimate treatment durability. Follow-up outcomes were also based on patient report obtained by telephone, SMS, or subsequent clinical contact, which introduces potential recall and social desirability bias. Finally, the association between symptom recurrence and periods of increased emotional stress was observational and does not establish that stress caused recurrence.

## Conclusions

This retrospective case series found that a four-week multimodal protocol was associated with rapid and clinically meaningful reductions in self-reported pain and improvements in health-related quality of life among men with persistent SCP. The conservative and non-destructive nature of the protocol preserves the option for surgical intervention when clinically indicated. However, the absence of a control group, the bundled nature of the intervention, and incomplete long-term follow-up prevent conclusions regarding causality, component-specific effects, or treatment durability. Restoration of neuromuscular control remains a plausible mechanism rather than a demonstrated explanation for the observed outcomes. Future prospective randomized trials with sham or active comparator groups, objective neurophysiological outcome measures, and longer follow-up are needed to confirm these findings and define the role of multimodal physiotherapy within the treatment algorithm for persistent SCP.
